# Reactive Oxygen Species Production and *Brugia pahangi* Survivorship in *Aedes polynesiensis* with Artificial *Wolbachia* Infection Types

**DOI:** 10.1371/journal.ppat.1003075

**Published:** 2012-12-06

**Authors:** Elizabeth S. Andrews, Philip R. Crain, Yuqing Fu, Daniel K. Howe, Stephen L. Dobson

**Affiliations:** 1 Department of Entomology, College of Agriculture, University of Kentucky, Lexington, Kentucky, United States of America; 2 Tropical Research and Education Center, University of Florida, Homestead, Florida, United States of America; 3 Gluck Equine Research Center, Department of Veterinary Science, University of Kentucky, Lexington, Kentucky, United States of America; Stanford University, United States of America

## Abstract

Heterologous transinfection with the endosymbiotic bacterium *Wolbachia* has been shown previously to induce pathogen interference phenotypes in mosquito hosts. Here we examine an artificially infected strain of *Aedes polynesiensis*, the primary vector of *Wuchereria bancrofti*, which is the causative agent of Lymphatic filariasis (LF) throughout much of the South Pacific. Embryonic microinjection was used to transfer the *w*AlbB infection from *Aedes albopictus* into an aposymbiotic strain of *Ae. polynesiensis*. The resulting strain (designated “MTB”) experiences a stable artificial infection with high maternal inheritance. Reciprocal crosses of MTB with naturally infected wild-type *Ae. polynesiensis* demonstrate strong bidirectional incompatibility. Levels of reactive oxygen species (ROS) in the MTB strain differ significantly relative to that of the wild-type, indicating an impaired ability to regulate oxidative stress. Following a challenge with *Brugia pahangi*, the number of filarial worms achieving the infective stage is significantly reduced in MTB as compared to the naturally infected and aposymbiotic strains. Survivorship of MTB differed significantly from that of the wild-type, with an interactive effect between survivorship and blood feeding. The results demonstrate a direct correlation between decreased ROS levels and decreased survival of adult female *Aedes polynesiensis*. The results are discussed in relation to the interaction of *Wolbachia* with ROS production and antioxidant expression, iron homeostasis and the insect immune system. We discuss the potential applied use of the MTB strain for impacting *Ae. polynesiensis* populations and strategies for reducing LF incidence in the South Pacific.

## Author Summary

Lymphatic filariasis (LF), the leading cause of morbidity in South Pacific regions, is caused by a filarial worm, *Wuchereria bancrofti*. Elimination of LF in the South Pacific requires an approach integrating both mass drug administration and strategies targeting the primary mosquito vector, *Aedes polynesiensis*. *Ae. polynesiensis* is naturally infected with *Wolbachia*, an endosymbiotic bacterium that is a focus of novel control strategies, due to its ability to affect mosquito reproduction and interfere with pathogen development. Artificial *Wolbachia* infections are associated with increased levels of reactive oxygen species (ROS), which can alter immune gene expression and inhibit dengue proliferation. Here, we describe the generation of an *Ae. polynesiensis* strain that has been artificially infected with *Wolbachia* from *Ae. albopictus*. The infection is stably maintained and causes conditional sterility when crossed with the wild-type. The artificially infected strain exhibits different ROS levels than the wild-type, indicating a decreased ability to regulate oxidative stress. The number of successfully developing infective stage filarial worms was reduced in the artificially infected strain. In addition, survival of the artificially infected strain was significantly lower than the wild-type. The artificially infected *Ae. polynesiensis* strain is discussed in relation to ongoing mosquito-borne disease control efforts.

## Introduction

Lymphatic filariasis (LF) affects 120 million people globally and has been a leading cause of morbidity in South Pacific regions [1]. An ongoing global campaign to eliminate LF relies upon mass drug administration (MDA) strategies. However, because of inherent issues associated with MDA, such as efficacy of antifilarial drugs and public compliance with drug regimens, an integrated approach that targets the vector has been suggested for the successful control of LF in some regions, such as the South Pacific.

*Aedes polynesiensis* is the primary vector of *Wuchereria bancrofti*, the filarial nematode that causes LF in the South Pacific [2]. This mosquito is naturally infected with *Wolbachia*, a maternally inherited endosymbiont that infects a broad range of invertebrates [3], [4]. *Wolbachia* infection in mosquitoes can induce cytoplasmic incompatibility (CI), a form of conditional sterility that results in early embryonic arrest when a *Wolbachia* infected male mates with an uninfected female or one harboring a different *Wolbachia* type [3]. Public health strategies under development are based upon manipulating *Wolbachia* induced CI in important mosquito species, either to suppress the population through releases of incompatible males or to harness CI as a gene-drive mechanism for spreading useful phenotypes, such as disease resistance, into a targeted population [5]–[7]. A recent proof of concept for this approach comes from a program in Australia that has successfully replaced an existing *Ae. aegypti* population with an artificially infected mosquito [8], [9].

*Wolbachia* infections, both natural and artificial, have been shown to interfere with pathogen development within the mosquito host. The presence of a naturally occurring *Wolbachia* infection in *Drosophila* protects flies from virus-induced mortality [10]. In mosquitoes, examples include the natural *Wolbachia* infection within *Culex quinquefasciatus*, which is associated with a significant reduction in West Nile virus dissemination and transmission rates [11]. Artificial *Wolbachia* infections have been observed to affect dengue, chikungunya, *Plasmodium* and filarial worms in *Ae. aegypti* [12], [13]. Formation of *Plasmodium falciparum* oocysts was inhibited in *Anopheles gambiae* that were somatically inoculated with *Wolbachia* [14].

Although the mechanism underlying pathogen interference is unknown, a possible explanation is the association between artificial *Wolbachia* infections and increased expression of key mosquito immune factors such as defensins, cecropins and Toll pathway genes [12], [13], [15]. In addition, recent studies have shown that artificial *Wolbachia* infections are associated with increased oxidative stress in the form of reactive oxygen species (ROS) [16], [17]. ROS are produced as the byproduct of aerobic metabolism [18] and can have detrimental effects on fecundity [19] and survival post blood meal [20]. However, a positive effect of high ROS levels is the inhibition of parasites within the mosquito host [21], [22]. Furthermore, elevated levels of ROS in *Ae. aegypti* are linked to the activation of the Toll immune pathway [17].

In this study, embryonic microinjection [23], [24] was used to introduce the *w*AlbB infection from *Ae. albopictus* into *Ae. polynesiensis*. Prior transfer of the *w*AlbB infection into *Ae. aegypti* induced strong CI in the resulting strain [25] and increased host viral resistance to dengue by increasing ROS levels and elevating expression of immune genes [15], [17]. In addition, the introduction of an artificial *Wolbachia* infection decreased filarial competence in *Ae. aegypti* [12]. We hypothesized that artificial introduction of the *w*AlbB infection into *Ae. polynesiensis* might facilitate a similar immunological response and reduce the intensity of filarial worm infection. Unlike other mosquito vector species, relatively little genomic information and molecular tools are available for *Ae. polynesiensis*, making examination of immune gene expression difficult. However, ROS measurement methods are a relatively robust indicator of immune system activation and have been applied to numerous species, including mosquito vectors [17], [22], [26], [27]. Here, we have compared ROS levels between the *Ae. polynesiensis* strains infected with different *Wolbachia* types. The results show an association between *Wolbachia* type and ROS levels. Comparisons of the *Ae. polynesiensis* strains show significant differences in their ability to support *Brugia pahangi* development. We discuss the results in relation to a possible interaction between *Wolbachia* infection type, ROS levels and filarial competency and the potential application to public health strategies targeting decreased LF incidence.

## Results

### Generation and characterization of the MTB strain

The MTB strain of *Ae. polynesiensis* was generated by microinjecting embryos of the aposymbiotic APMT strain with cytoplasm from naturally superinfected *Ae. albopictus* embryos (HOU strain). The mosquito strains used in the injection experiment are listed in Table 1, and the outcome of one injection experiment is shown in Table 2. Of 134 injected APMT embryos, 17 G_0_ females and 12 G_0_ males survived to adulthood, seven and six of which were infected with *Wolbachia*, respectively.

**Table 1:** Mosquito strains.

Strain Name	Mosquito Species	*Wolbachia* Type
HOU	*Ae. albopictus*	*w*AlbA/*w*AlbB
APM	*Ae. polynesiensis*	*w*PolA
APMT	*Ae. polynesiensis*	none
MTB	*Ae. polynesiensis*	*w*AlbB

**Table 2:** Survival of microinjected *Ae. polynesiensis* embryos for each life stage and the resulting *Wolbachia* infection rates in the G_0_ adults.

G_0_ Survival				G_0_ Infection Rate (infected/total)	
Hatch % (larvae/eggs)	Pupation % (pupae/larvae)	Eclosion (adults/pupae)	Sex Ratio % (female/total)	Female	Male
24.6% (33/134)	93.9% (31/33)	93.5% (29/31)	58.6% (17/29)	41.2% (7/17)	50.0% (6/12)

The seven PCR positive G_0_ females were screened for specific infection type (Table 3). A G_0_ female infected with both *w*AlbA and *w*AlbB was chosen for further selection. Her daughters (G_1_) had a 60% infection rate, with the majority of females single-infected with *w*AlbB only. PCR testing and selection of the subsequent generations were unable to sustain the superinfection. Thus, the resulting MTB strain is infected with *w*AlbB only. Using PCR-guided selection, infected females were continuously outcrossed with APMT males until G_6_. Beginning at G_7_, MTB females were mated with MTB males. Subsequent to G_7_, periodic testing of the MTB strain confirmed that the infection is stable and maternal inheritance rates remain at 100% (data not shown).

**Table 3:** *Wolbachia* infection following the generation of the MTB strain.

		Female Infection Type					Male Infection Type					
Generation	Mate Type	AB	A	B	Ø	Female IR (%)	AB	A	B	Ø	Male IR (%)	Overall IR (%)
G_0_	APMT	4	0	3	10	41.2	6	0	0	6	50	44.8
G_1_	APMT	1	0	5	4	60	1	0	0	3	25	50
G_2_	APMT	0	0	9	0	100	-	-	-	-	-	100
G_3_	APMT	0	0	5	0	100	-	-	-	-	-	100
G_4_	APMT	0	0	23	2	92	0	0	3	0	100	92.9
G_5_	APMT	0	0	17	0	100	0	0	1	0	100	100
G_6_	APMT	0	0	20	0	100	-	-	-	-	-	100
G_7_	MTB	0	0	10	0	100	0	0	10	0	100	100

IR = infection rate. AB = superinfected with *w*AlbA*/w*AlbB, A = single infected with *w*AlbA, B = single infected with *w*AlbB. Males were not tested for *Wolbachia* infection every generation.

Crosses were performed to examine for CI. The results demonstrate bidirectional incompatibly between APM and MTB. High egg hatch was observed in crosses between similar males and females (Table 4). In contrast, few eggs hatched in reciprocal crosses between the strain types. Incompatible crosses were significantly different from the controls (H = 12.5, df = 3, p = 0.0059).

**Table 4:** Egg hatch rates resulting from crosses.

		Infection Types			
Expected CI Type	Cross (FxM)	Female	Male	Percent Hatch Rate (Mean ± SD)	Eggs Scored (#)
Incompatible	APM×MTB	*w*PolA	*w*AlbB	0.0±0.0, A	1960
	MTB×APM	*w*AlbB	*w*PolA	0.0±0.1, A	1581
Compatible	APM×APM	*w*PolA	*w*PolA	71.9±29.6, B	1276
	MTB×MTB	*w*AlbB	*w*AlbB	62.7±29.7, B	1164

Different letters following the data indicate significant differences (Wilcoxon, q = 1.96, p<0.05).

### ROS levels in *Ae. polynesiensis* strains

**Figure 1:**
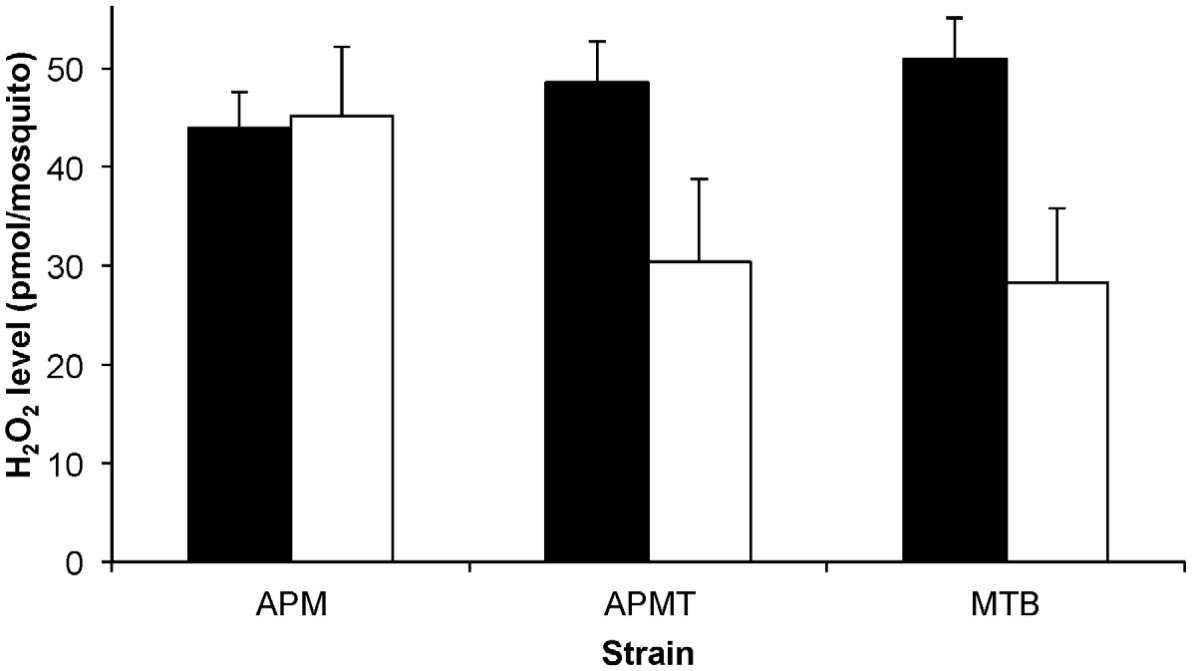
ROS levels in *Ae. polynesiensis* strains. The concentration of H_2_O_2_ was measured in APM, APMT and MTB after feeding on sucrose (black) and 24 hours after a blood meal (white). Sucrose fed MTB had significantly higher levels of ROS than APM (ANOVA, p<0.05). Blood feeding was associated with a significantly reduced ROS level in APMT and MTB (ANOVA, p<0.05) but not APM (p = 0.75). After blood feeding, APM maintained higher ROS levels than APMT and MTB (Tukey, p<0.05), which were equivalent (p = 0.9). The data shown are the means of five replicates.

ROS levels can be significantly affected in mosquitoes that are artificially infected with *Wolbachia* [17]. To examine for a *Wolbachia-*mediated effect in *Ae. polynesiensis*, we compared ROS levels in young adult females of MTB, APM and APMT. In addition to examining females fed sucrose only, we provided females with a blood meal to examine for an effect of blood feeding on ROS levels, which has been observed in prior studies [22], [28]. The results (Figure 1) show that blood meal status had a significant effect on ROS levels, but only for the strains in which *Wolbachia* had been manipulated (*i.e*., not in the naturally infected strain). Specifically, a model with strain and blood meal status as factors and ROS level as the variable was significant (GLM: χ^2^ = 38.2, df = 5, p<0.0001). Blood meal status was significant (GLM: χ^2^ = 27.2, df = 1, p<0.0001), while the overall strain effect was not significant (GLM: χ^2^ = 5.12, df = 2, p = 0.07). A significant interactive effect was observed for strain×blood meal status (GLM: χ^2^ = 19.3, df = 2, p<0.0001). Following a blood meal, ROS levels in the APM strain remained similar to those observed in sucrose fed females (ANOVA, F_1,8_ = 0.11, p = 0.75). However, significant decreases in ROS levels are observed for blood fed females of APMT (ANOVA, F_1,8_ = 19.09, p<0.05) and MTB (ANOVA, F_1,8_ = 35.08, p<0.001). Post hoc Tukey HSD tests determined that after blood feeding, APM had significantly higher levels of ROS than APMT (p<0.05) and MTB (p<0.05), the latter of which were equivalent (p = 0.9).

### Comparative filarial susceptibility and survivorship

**Figure 2:**
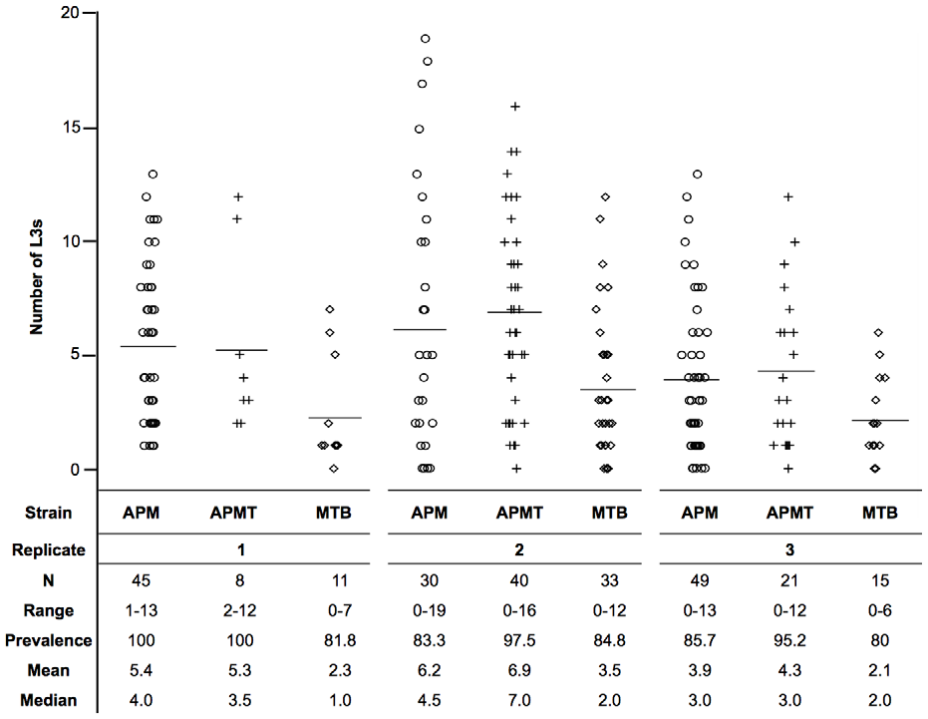
Mean number of L3s within *Ae. polynesiensis* strains. APM, APMT and MTB were given a *Brugia pahangi* infected blood meal. After ten days, mosquitoes were dissected and the number of infective stage L3s was counted. The MTB strain had a significantly lower worm load (p<0.0001) than APM and APMT, which were equivalent (p = 0.47). Despite significant variation between replicates (p<0.0001), the relationship between strains (APM = APMT>MTB) was consistent across the three replicates (p = 0.77). Each symbol represents a single mosquito. Lines represent mean values.

Prior studies have shown that changes in ROS levels can be detrimental to *Plasmodium* development in *Anopheles gambiae* [21], [22], [27] and that artificial *Wolbachia* infection can affect filarial worm development in *Ae. aegypti* [12]. Therefore, the number of infective stage filarial worms were compared following a *Brugia*-infected blood meal (Figure 2). MTB had significantly lower worm loads relative to both APM (χ^2^ = 53.3, df = 1, p<0.0001) and APMT (χ^2^ = 44.2, df = 1, p<0.0001). Equivalent worm loads were observed with APM and APMT (χ^2^ = 0.52, df = 1, p = 0.47).

**Figure 3:**
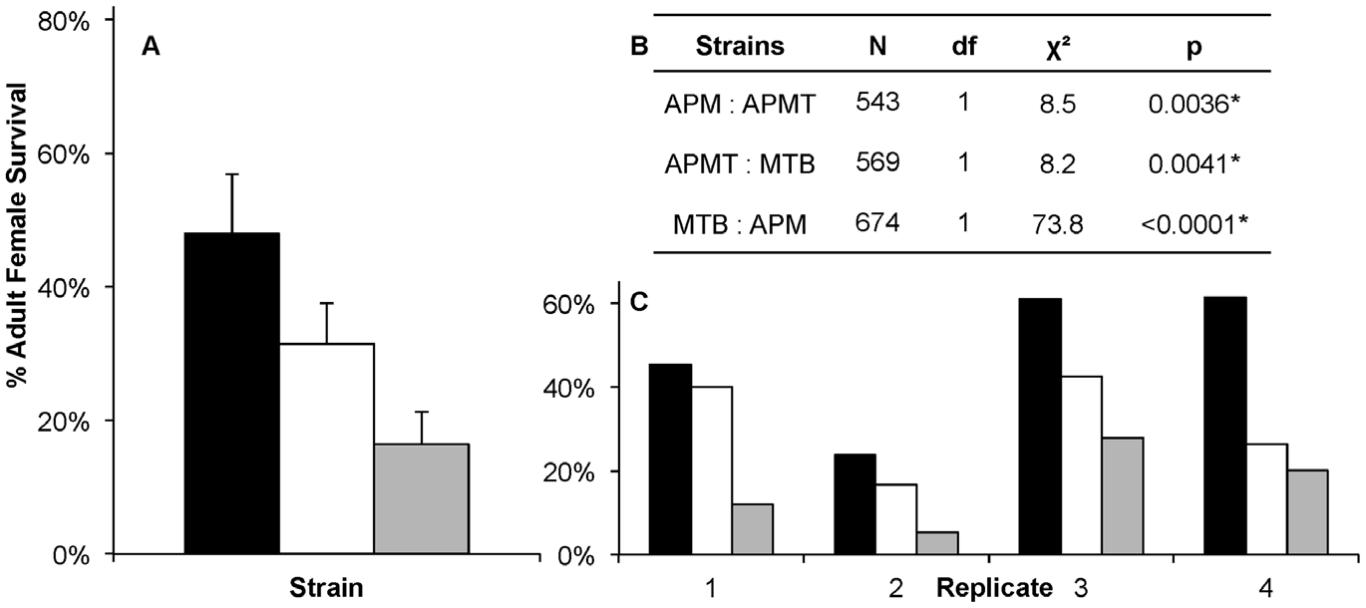
Survivorship of *Ae. polynesiensis* strains after a *Brugia*-infected blood meal. Mean survivorship of APM (black), APMT (white) and MTB (grey) ten days after feeding on a *Brugia pahangi*-infected blood meal. Contrast comparisons demonstrate significant differences in survivorship for each pair-wise comparison between strains. A) The mean survival of the four replicates. Error bars represent standard error. B) Contrast comparisons. C) The relationship between strains (APM>APMT>MTB) was consistent across all four replicates (GLM: χ^2^ = 6.27, df = 6, p = 0.39). For all tests, α = 0.05.

Observations during competency experiments suggested a difference in strain survivorship after feeding on *Brugia*-infected blood. Therefore, a formal experiment was conducted to compare survivorship. Significant differences were observed in survivorship between strains fed *Brugia*-infected blood (GLM: χ^2^ = 119.6, df = 11, p<0.0001, Figure 3). There were significant differences between replications (GLM: χ^2^ = 28.5, df = 3, p<0.0001) and strain (GLM: χ^2^ = 73.79, df = 2, p<0.0001). Despite the variation between replicates, the pattern between strains remained consistent (GLM: strain×replicate, χ^2^ = 6.27, df = 6, p = 0.39; Figure 3C).

**Figure 4:**
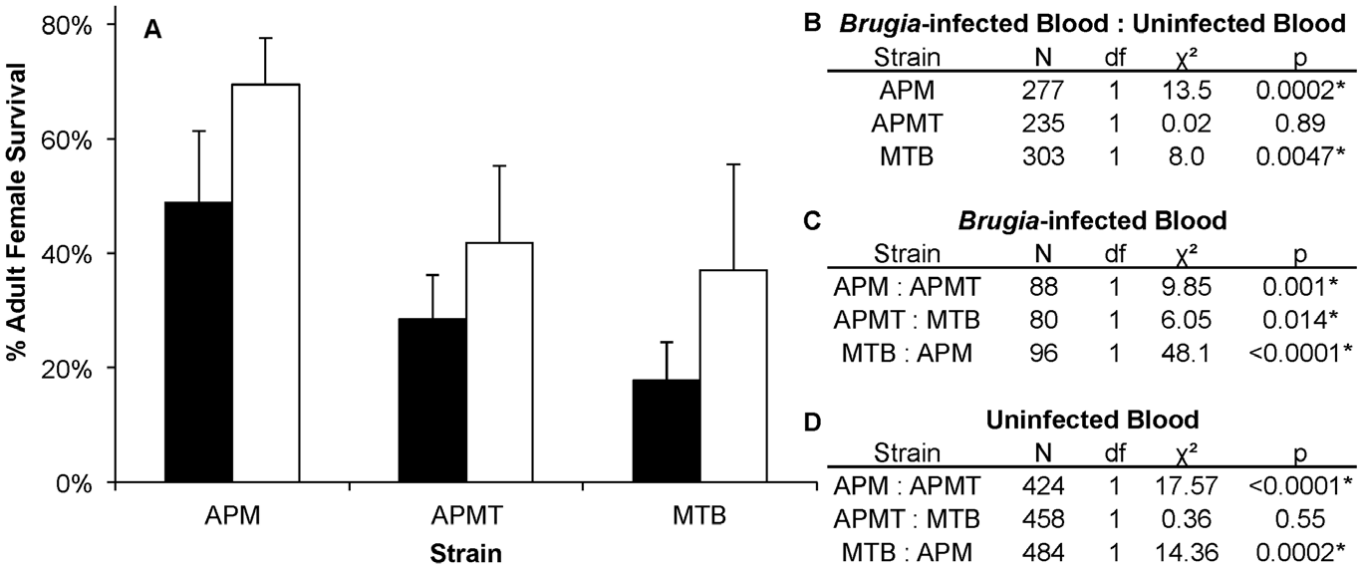
Survivorship of *Ae. polynesiensis* strains after feeding on *Brugia*-infected blood vs. uninfected blood. A) Mean survivorship of APM, APMT and MTB ten days after feeding on *Brugia pahangi*-infected blood (black) vs. uninfected blood (white). The presence of *Brugia pahangi* in the blood meal was associated with decreased survivorship for APM (p<0.05) and MTB (p<0.05). APMT survivorship was independent of blood meal type (p = 0.89). Error bars represent standard error for three replicates. B) Contrast comparisons for strains fed *Brugia*-infected and uninfected blood. C) Contrast comparisons for strains fed *Brugia*-infected blood. D) Contrast comparisons for strains fed uninfected blood.

To examine for a role of the *Brugia* parasite in the different survivorship, the strains were compared when fed on blood either with or without *Brugia* (Figure 4). The results show that, in general, each of the strains experienced lower survivorship when fed *Brugia*-infected blood, relative to uninfected blood (GLM: χ^2^ = 14.3, df = 1, p = 0.0002). Similar to the pattern observed in the preceding experiment, APM was longer lived than APMT (GLM: χ^2^ = 9.85, df = 1, p<0.05), which was longer lived than MTB (GLM: χ^2^ = 6.05, df = 1, p<0.05). There was no significant interactive effect for blood meal type×strain (GLM: χ^2^ = 4.5, df = 2, p = 0.1).

**Figure 5:**
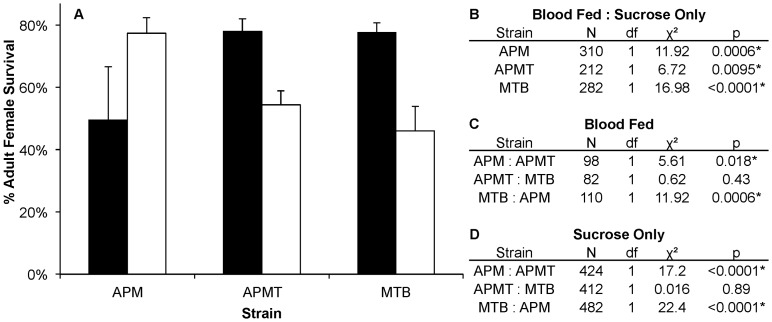
Survivorship of *Ae. polynesiensis* strains after blood feeding vs. sucrose only. A) Mean survivorship of APM, APMT and MTB ten days after blood feeding (white) vs. sucrose only (black). Survival increased in APMT (p<0.05) and MTB (p<0.05) when fed sucrose only. However, decreased survival was observed in APM when not given a blood meal (p<0.05). Error bars represent standard error of two replicates. B) Contrast comparisons for strains fed blood or sucrose only. C) Contrast comparisons for strains fed blood. D) Contrast comparisons for strains fed sucrose only.

Comparing females fed either blood or sucrose only (Figure 5), the pattern was similar to that observed for ROS levels (Figure 1). The two artificially infected strains experienced significantly reduced survival following a blood meal. Specifically, APMT (GLM: χ^2^ = 6.72, df = 1, p<0.05) and MTB (GLM: χ^2^ = 16.98, df = 1, p<0.05) were longer lived when fed sucrose only. In contrast, APM females were longer lived following a blood meal (GLM: χ^2^ = 11.92, df = 1, p<0.05).

## Discussion

### A novel *Wolbachia* infection in *Aedes polynesiensis*

Embryonic microinjection was used to transfer the *w*AlbB infection from *Ae. albopictus* to *Ae. polynesiensis*. PCR assays show that the infection is stable, with high maternal transmission. Overall mosquito survival after microinjection was high as compared to previous studies, which observed survival rates of less than 5% [24], [29]. Prior crossing experiments found that *w*PolA [30] and *w*AlbB [31] cause cytoplasmic incompatibility when crossed with different *Wolbachia* types. Consistent with expectations, crosses between APM and MTB were bidirectionally incompatible. Although superinfected cytoplasm was injected, only the *w*AlbB *Wolbachia* infection was established. The separation of superinfected *Wolbachia* types after microinjection is consistent with prior reports [31]–[34] and may result from different infection levels of *w*AlbB versus *w*AlbA within superinfected *Ae. albopictus* [35].

### ROS levels and *Wolbachia* infection

The differing ROS levels observed in the artificially infected *Ae. polynesiensis* strains is similar to that of previous reports, which have shown differing ROS levels resulting from transinfection with *w*AlbB *Wolbachia* infection in both adult female *Ae. aegypti* [17] and in an *Ae*. *albopictus* cell line [16]. Multiple cellular pathways, including iron metabolism and immunity, influence ROS levels in mosquitoes [17], [36]. For example, H_2_O_2_ can be catalyzed via the Fenton reaction, along with excess labile iron [36]. Prior studies have demonstrated *Wolbachia*-produced bacterioferritin can scavenge labile iron, with the potential for iron competition between *Wolbachia* and host [16], [37]–[44]. Unfortunately, testing of specific hypotheses is limited at present, since many of the genomic and molecular tools available for better studied mosquito species (e.g., *Ae. aegypti*) are not yet available with *Ae. polynesiensis*. Our results provide additional motivation for developing such tools and methods.

Particularly intriguing is the previously reported ability of *Wolbachia* to upregulate dual oxidase (DUOX), which may influence the observed variation in ROS levels. A key component of innate immunity, the DUOX transmembrane protein is involved in ROS generation [26], [45]–[47] and can be increased if an artificial *Wolbachia* infection is recognized as foreign by its mosquito host [17]. Imbibing a blood meal can cause significant oxidative stress due to heme released from the degradation of hemoglobin, which can have pro-oxidant and cytotoxic effects when not bound to regulatory proteins [48]. An ability to maintain homeostasis despite this massive influx of iron resulting from blood feeding is important to the evolutionary success of hematophagous insects [36]. In addition, the *Wolbachia* genome retains genes for heme biosynthesis [49]–[51], and naturally occurring *Wolbachia* infections have been shown to buffer iron flux in insects, allowing iron homeostasis despite large influxes and limiting the deleterious effect of labile iron [37], [38]. In the wild type APM strain, no overall variation in ROS levels were observed following a blood meal. In contrast, a significant decrease in ROS levels was observed in the artificially infected MTB and aposymbiotic APMT strains following a blood meal. The reintroduction of *w*AlbB did not restore MTB to the homeostasis phenotype observed in the wild-type APM strain, indicating that not all *Wolbachia* types are equivalent and that the *w*PolA infection in *Ae. polynesiensis* represents an evolved symbiosis.

Whole mosquitoes were examined in this study, which may mask a tissue-specific effect. For example, mitochondrial generation of H_2_O_2_ was reduced in flight muscles after blood feeding in *Ae. aegypti* [52], and ROS levels were elevated in *An. gambiae* hemolymph following a blood meal [22]. In *Ae. aegypti,* blood feeding was associated with a significant decrease in ROS levels in midgut tissue through activation of a heme-mediated protein kinase C pathway [28]. While beyond the scope of our experimental design, our results encourage additional experiments designed explicitly to examine ROS in specific tissues in an effort to better understand the role of *Wolbachia* in influencing iron metabolism and the mosquito immune system.

### Filarial worm survival

The number of infective stage filarial worms that developed within *Ae. polynesiensis* differed significantly between the strains. Specifically, the wild-type *Wolbachia* infected APM and its aposymbiotic counterpart (APMT) had similar numbers of successfully developing, infective L3 worms. This is comparable to the result of previous studies in which the removal of naturally occurring *Wolbachia* in *Ae. pseudoscutellaris* had no effect on the mean number of worms [53]. In contrast, significantly lower worm loads were observed in the artificially infected MTB compared to both the naturally infected and aposymbiotic strains. The latter is consistent with a prior experiment in which *Wolbachia* was artificially introduced into *Ae. aegypti* [12]. In the prior study, significantly lower *B. pahangi* numbers were observed in the artificially infected strain, relative to the naturally uninfected *Ae. aegypti*. Using the substantial genomic information available for *Ae. aegypti*, the authors speculated upon an association between an observed constitutive up-regulation of immune genes and an observed inhibition of filarial worm development. With the future development of additional genetic tools for *Ae. polynesiensis*, a similar approach can be used downstream to examine for an impact of artificial *Wolbachia* infection.

Artificial *Wolbachia* infections can detrimentally affect the fitness of their hosts [54]–[56]. In this study, reduced survival was observed for females with artificial *Wolbachia* infection types when fed on infected or uninfected blood. The decreased number of L3 filarial worms within MTB may be due, at least in part, to a reduced ability of MTB females to tolerate filarial worm infections and their premature deaths prior to dissection assays. The observed ROS variation is an additional potential explanation for the observed variation in filaria development. Recent studies show that changes in ROS levels can affect pathogen development. In *An. gambiae,* high levels of ROS were associated with increased melanotic encapsulation of *Plasmodium* parasites [22], [27]. In *Ae. aegypti,* increased ROS expression is associated with induction of the Toll pathway, which mediates the expression of antimicrobial peptides and antioxidants to balance oxidative stress and is associated with reduced dengue virus titer [17]. In addition, ROS generated independent of the mosquito immune system by the native microflora have also been found to negatively affect development of *Plasmodium* parasites [21]. It is also relevant to highlight the importance of iron to filarial worm development [57], [58]. If the artificial *Wolbachia* infection in *Ae. polynesiensis* were to affect the regulation of iron, as previously discussed, then filarial worm development and survival may be affected in the MTB strain. However, a simple direct association with overall ROS levels cannot explain the pattern of differential filarial worm development that was observed here, since the overall ROS levels were lower in MTB, relative to wild type mosquitoes. Furthermore, the lower ROS levels observed in the aposymbiotic APMT strain was not observed to be associated with reduced filarial development.

The variation observed between experimental replicates is not unexpected and is similar to prior reports [59]–[61]. Possible reasons for this variability include differences in sausage casing thickness, blood quality, and additional factors that affect mosquito feeding and microfilariae. Importantly and directly related to the study design, regardless of the variation between replications, the observed differences between strains remained the same (Figure 3C).

### Survivorship and blood meal status

Filarial worm infections in mosquitoes are not benign. They can cause damage to the midgut and flight muscles [62], [63], sometimes affecting flight behavior [64]. Increasing the number of worms in an infected blood meal decreases mosquito survival rates [65]. Our results confirm the detrimental nature of filarial worms present in a blood meal, where a general reduction in survival was observed for all of the examined strains (Figure 4).

Blood contains an important nutritional component for adult mosquitoes [66]–[68] so it is not surprising that reduced survival was associated with blood deprivation of wild-type APM mosquitoes. However, this pattern was reversed in the aposymbiotic APMT and artificially infected MTB strains, where we observed a significant reduction in survivorship after blood feeding as compared to sucrose fed mosquitoes (Figure 5). As described above, this may reflect an evolved mutualism between the *w*PolA infection and *Ae. polynesiensis*, since increased survival of blood fed females is adaptive for both the anautogenous mosquito and the maternally inherited *Wolbachia* infection.

The observed pattern of decreased survivorship was similar to the pattern of H_2_O_2_ levels (Figure 1), suggesting an association with ROS homeostasis. Similar to our results, a recent study observed a link between decreased ROS levels and the proliferation of gut bacteria, which can be detrimental to the survival of the mosquito [27], [28]. In *An. gambiae* that were artificially infected with *Wolbachia*, no fitness effect was observed until a blood meal was taken. The authors proposed that this virulence could be due to modulated ROS levels and proliferation of gut bacteria within the mosquito following a blood meal [14].

### Conclusions

In this study we were able to successfully infect *Ae. polynesiensis* with an artificial *Wolbachia* type that is stably maintained and causes bidirectional cytoplasmic incompatibility when crossed with the wild-type strain. We observed that the removal of *Wolbachia* and subsequent introduction of a novel *Wolbachia* type into *Ae. polynesiensis* affected host physiology and filarial worm development. We observed significant effects on ROS production both before and after blood feeding. The artificially infected mosquitoes varied also in their ability to support filarial worm development. Decreased survival was observed for blood fed mosquito strains that were cleared of their natural *Wolbachia* infection. The results encourage additional investigation into the specific physiological mechanisms affected by the artificial *Wolbachia* infection in *Ae. polynesiensis*. In addition, the findings presented here lend support for additional experiments with the human parasite, *W. bancrofti*. Although *B. pahangi* is a commonly used model system, it is important to determine if similar inhibitory effects can be observed against *W. bancrofti*. However, as there is no animal model, the latter will require transporting the transinfected mosquitoes to an endemic area or importing infected blood.

The observed experimental outcomes suggest applied strategies to impact filarial worm transmission by *Ae. polynesiensis* in the South Pacific. Specifically, the bidirectional incompatibility occurring in crosses of MTB and the wild-type provides a potential means to reduce the population of this important vector [2]. Another avenue of control is to purposely replace the existing population with MTB. The latter strategy would be similar to ongoing work in Australia against dengue transmission by *Ae. aegypti* [8], [9]. However, since MTB is bidirectionally incompatible with the wild type population, one would not expect CI to drive the spread of *Wolbachia*. Instead, the strategy would be suppression followed by female releases to establish the new infection type [69]. Furthermore, the decreased MTB survivorship observed in blood fed females and their reduced filarial worm development are consistent with the phenotype desired for a strategy in which the indigenous *Ae. polynesiensis* population is replaced with a strain less able to transmit filarial worms.

## Materials and Methods

### Ethics statement

This study was performed in strict accordance with the recommendations in the Guide for Care and Use of Laboratory Animals of the National Institutes of Health. The protocol was approved by the Institutional Animal Care and Use Committee (IACUC) of the University of Kentucky (Protocol number: 00905A2005).

### Mosquito strains

*Aedes albopictus* (HOU) and an aposymbiotic *Ae. polynesiensis* strain (APMT) were used as *Wolbachia* donor and recipient, respectively. The HOU donor strain is naturally super-infected with two *Wolbachia* types, *w*AlbA and *w*AlbB [70]. The recipient strain, APMT, was generated by tetracycline treatment of the APM strain [2] and has been maintained for >30 generations in the laboratory. The wild-type APM strain is single infected with *w*PolA and exhibits a 100% infection rates in wild populations [30], [71] (Table 1). APM was used in crossing tests to characterize the newly generated strain, MTB.

Unless otherwise specified, mosquitoes were maintained using standard insectary conditions at 28±2°C, 75±10%RH, and a photoperiod of 18∶6 h (L∶D). Larvae were reared in optimal conditions, at low density in excess of 6% liver powder solution (MP Biomedicals, LLC, Solon, OH), until pupation. Adult mosquitoes were provided with a 10% sucrose solution *ad libitum,* and a blood meal was given once a week with anesthetized mice.

### Embryonic microinjection

Collection, preparation and microinjection of embryos were based upon successful techniques used for previous mosquito transfections [23], [31]. Injection needles were prepared using quartz glass capillaries with an outer diameter (OD) of 1.00 mm, an inner diameter (ID) of 0.70 mm, and a length of 7.5 cm (QF100-70-7.5; Sutter Instrument Co., Novato, CA). Needles were beveled at a 15° angle using a micropipette beveler, model BV-10 (Sutter Instrument Co., Novato, CA). Microinjection was done using an Olympus IX70 inverted microscope (Olympus Co., Tokyo, Japan) at ×200 magnification.

Blood-fed APMT females were held in *Drosophila* vials (Fisher Scientific) containing wet germination paper (Anchor Paper Co., Saint Paul, MN) and allowed to oviposit. Recipient embryos (APMT) to be injected were collected, aligned on wet germination paper, briefly desiccated and covered with water-saturated halocarbon 700 oil (Sigma-Aldrich Co.). Donor HOU embryos were treated similarly, but not desiccated.

Cytoplasm was withdrawn from the posterior of donor HOU embryos and injected using an IM 300 microinjector (Narishige Scientific, Tokyo, Japan) into the posterior of the recipient APMT embryos. Recipient embryos were injected up to 90 minutes post-oviposition. After injection, the embryos were incubated under standard conditions for approximately 40 minutes. Injected embryos were removed from oil and transferred to wet germination paper, where they were allowed to develop for 5 days. The eggs were hatched (G_0_) and reared using standard maintenance conditions.

### Rearing and selection of microinjected lines

Females of the parent generation (G_0_) were isolated as virgins and mated with APMT males, yielding a new strain named MTB. After oviposition, G_0_ females and males were assayed for both presence of *Wolbachia* infection and type using PCR (see below)(Table 3). Females that were negative for *Wolbachia* were discarded along with their progeny. Daughters (G_1_) from infected G_0_ females were isolated as virgins and outcrossed with APMT males. All G_1_ females that oviposited were tested for *Wolbachia* infection by PCR. PCR-guided selection was performed for 6 generations (G_1_–G_6_) (Table 3). At G_7_ the MTB strain was closed (i.e. not outcrossed with APMT males, but crossed with MTB males), and PCR was used to monitor the frequency of infection periodically through the following generations.

### Infection status testing via PCR amplification

All infection types were confirmed using *Wolbachia* specific primers and PCR. Adults were homogenized in 100 µl of buffer containing 10 mM Tris-HCl, 1 mM EDTA and 50 mM NaCl using a Mini-beadbeater (BioSpec Products, Inc., Bartlesville, OK), boiled for 5 minutes and centrifuged at 14,000 rpm for 5 minutes. Two µl of supernatant were used for each PCR reaction. PCR reactions were amplified in 50 mM KCl, 20 mM Tris-HCl (pH 8.4), 1.5 mM MgCl_2_, 0.25 mM dNTPs, 0.5 mM primers and 1 U Taq DNA polymerase in a total volume of 25 µl. *Wolbachia* infection in all strains was confirmed using general *Wolbachia* primers 438F (5′CAT ACC TAT TCG AAG GGA TAG-3′) and 438R (5′AGC TTC GAG TGA AAC CAA TTC-3′) and PCR cycling conditions of 94°C 2 minutes, 39 cycles of 94°C for 30 seconds, 55°C for 45 seconds and 72°C for 1 minute 30 seconds, followed by a final extension temperature of 72°C for 10 minutes. Infection type of all strains was confirmed using A-clade (136F and 691R) or B-clade (81F and 522R) specific primers [72]. PCR cycling conditions were 94°C for 4 minutes, followed by 35 cycles of 94°C for 1 minute, 48°C (A) or 55°C (B) for 1 minute and 72°C for 1 minute and a final extension temperature of 72°C for 10 minutes.

### Cytoplasmic incompatibility

Similarly aged egg papers from APM and MTB were hatched concurrently in dilute liver powder solution (∼0.6 g/L). One hundred first instar larvae were moved into a rearing container and fed optimally until pupation. Pupae were isolated in individual test tubes to ensure virginity. After eclosion, 20 virgin adults were introduced into a crossing cage at a 1∶1 sex ratio and allowed to mate. A full factorial crossing design between APM and MTB was implemented, and four replicates were performed for each cross (Table 4). An oviposition cup was available to females in each crossing cage for one week, after which the cup was removed. Eggs remained hydrated and were allowed to mature for 10 days. Egg papers were removed from the oviposition cups and hatched by submersion for two days in dilute liver powder solution. All eggs were examined by microscope to determine the total number of eggs and the proportion hatched as indicated by the position of the operculum. The normality of the hatch rate data was analyzed using a Shapiro-Wilkes test (JMP, SAS Institute, Cary, NC). A Kruskal-Wallis test was used to determine overall significance and post-hoc Wilcoxon tests were used for pairwise comparisons of hatch rates between crosses.

### Determination of ROS in the whole mosquito

To determine ROS levels in mosquitoes fed sucrose only, whole bodies of seven-day-old APM, APMT and MTB were collected in 150 µl of 1× PBS containing 2 mg/ml of the catalase inhibitor 3-amino-1, 2, 4-trizole. To determine ROS levels in blood fed mosquitoes, six-day-old APM, APMT and MTB were provided with a blood meal from an anesthetized mouse. Twenty-four hours after blood feeding, the midgut was dissected from the mosquito and the blood bolus was flushed from the midgut using 1× PBS with catalase inhibitor. Mosquito carcasses and midgut tissues were collected in 1× PBS with catalase inhibitor.

For both treatments, samples were homogenized then centrifuged for 5 minutes at 10,000 g. The supernatant was filtrated through a 10 K molecular weight cutoff spin filter (Corning SpinXUF; Corning Incorporated Life Sciences). The elution was collected and tested using a Hydrogen Peroxide Assay kit (BioVision) following manufacturer's instructions. The fluorescence intensity was detected with Excitation/Emission 544/590 using a fluorescence microplate reader (Fluoroskan Ascent FL, Thermolabsystems). Five biological replicates, with three females for each strain were used for each treatment. A general linearized model with a normal distribution was used to determine if ROS levels differed between strain, feeding status or strain×feeding status. The sucrose treatment and the blood treatment were analyzed using separate ANOVAs with post hoc Tukey HSD comparisons.

### Filarial susceptibility testing

Three replicates were performed to test for relative filarial susceptibility between strains. *Brugia pahangi*-infected dog blood was provided from the NIH/NIAD Filariasis Research Reagent Resource Center at the University of Georgia. Egg papers for APM, APMT and MTB were hatched concurrently and reared under standard maintenance conditions. Adult female mosquitoes were anesthetized using chloroform, and 75–90 mosquitoes were placed into cages. They were provided with a 10% sucrose solution and given 3 days to acclimate to the cage. Females aged 3–5 days were sucrose starved for 6 hours prior to blood feeding. They were given a *Brugia*-infected blood meal (10 microfilariae/µl) using sausage casing and a Hemotek membrane feeding system (Discovery Workshops, Accrington, UK) that maintained the blood at 37°C. All mosquito strains were allowed access to blood for 2 hours.

After feeding, females were allowed to rest for one hour before sorting. All mosquitoes were briefly anesthetized using chloroform and observed under a microscope for presence of a blood bolus. Blood fed and non-blood fed females were placed into separate cages. Ten days after feeding, surviving blood fed females were anesthetized on ice and dissected in sterilized Hank's balanced salt solution (Sigma-Aldrich). Individual mosquitoes were examined for L3 parasites by microscopy. The total number of filarial worms in each mosquito was recorded.

To determine whether worm load data were normal, a Shapiro-Wilkes test was used (JMP, SAS Institute, Cary, NC). We built a general linearized model with a Poisson distribution to determine if mean worm load differed across replicates or between strains. Post-hoc contrasts were used to compare worm loads between strains. To correct for multiple comparisons we used the Benjamini-Hochberg correction with an α value of 0.05 [73].

### Mosquito survivorship post blood feeding

Mosquito rearing and blood feeding methods were the same as those described in “filarial susceptibility testing.” We recorded the number of mosquitoes alive and dead ten days after feeding on different blood meal types to compare differences in survival between APM, APMT and MTB. Three separate experiments were performed: 1) comparisons between strains fed on *Brugia*-infected blood only, 2) comparisons between mosquitoes fed uninfected and *Brugia*-infected blood meals, and 3) comparisons between mosquitoes that were blood fed or fed sucrose only.

For each of the above experiments, we built a general linearized model with a binomial distribution to determine if survivorship at day 10 differed between replicate, strain and blood meal type (JMP, SAS Institute, Cary, NC). Post-hoc contrasts were used to compare survivorship between strains. To correct for multiple comparisons we used the Benjamini-Hochberg correction with an α value of 0.05 [73].
